# Lipopolysaccharides in diazotrophic bacteria

**DOI:** 10.3389/fcimb.2014.00119

**Published:** 2014-09-03

**Authors:** Rodrigo V. Serrato

**Affiliations:** Setor Litoral, Universidade Federal do ParanáMatinhos, Brasil

**Keywords:** lipopolysaccharide, plant-bacterium interaction, nitrogen-fixation, associative diazotrophs, nodulating diazotrophs

## Abstract

Biological nitrogen fixation (BNF) is a process in which the atmospheric nitrogen (N_2_) is transformed into ammonia (NH_3_) by a select group of nitrogen-fixing organisms, or diazotrophic bacteria. In order to furnish the biologically useful nitrogen to plants, these bacteria must be in constant molecular communication with their host plants. Some of these molecular plant-microbe interactions are very specific, resulting in a symbiotic relationship between the diazotroph and the host. Others are found between associative diazotrophs and plants, resulting in plant infection and colonization of internal tissues. Independent of the type of ecological interaction, glycans, and glycoconjugates produced by these bacteria play an important role in the molecular communication prior and during colonization. Even though exopolysaccharides (EPS) and lipochitooligosaccharides (LCO) produced by diazotrophic bacteria and released onto the environment have their importance in the microbe-plant interaction, it is the lipopolysaccharides (LPS), anchored on the external membrane of these bacteria, that mediates the direct contact of the diazotroph with the host cells. These molecules are extremely variable among the several species of nitrogen fixing-bacteria, and there are evidences of the mechanisms of infection being closely related to their structure.

## Introduction

With the exception of water, nitrogen is the most limiting compound for plant growth and production. Despite being found in abundance in the Earth's atmosphere as molecular dinitrogen (N_2_), it is unavailable to plants which can only use reduced forms of this element, such as ammonia (NH_3_). A very specialized group of prokaryotes, named diazotrophs, are able to carry out the conversion of gaseous N_2_ into ammonia in a process known as biological nitrogen fixation (BNF), discovered by Martinus Beijerinck in 1901. The BNF process had a major breakthrough in the early 1970's during the oil crisis, when the price of petroleum rose vertiginously, thus affecting the prices of production and transportation of chemical fertilizers. With the aid of BNF plants can readily assimilate NH_3_ to produce important biomolecules such as proteins, nucleic acids, ATP, chlorophyll, among others. Diazotrophic microorganisms include aquatic cyanobacteria and free-living bacteria in soil, but a variety of these prokaryotes form associative relationships with plants, and most interestingly, a few have developed an interdependent symbiosis with their hosts, especially legumes, in which specialized structures (nodules) where BNF takes place are formed in the roots. The infection process in which soil bacteria interact with their plant hosts is very complex and yet not fully understood. In the case of nodulating diazotrophs, it is known that exopolysaccharides (EPS) and lipochitooligosaccharides (LCO) that are released in the surrounding microbe environment have paramount importance in all different stages of infection, as well as on the stimulation of cell division in the plant causing the nodule to form in legumes. In this mini-review, however, the focus is on the role of lipopolysaccharides (LPS) during the diazotroph-plant interaction, since these glycoconjugates are present on the outer membrane of these microorganisms and create an intimate “face-to-face” interaction between plant root-cells and nitrogen-fixing bacteria. Knowledge gained in the understanding of the molecular basis for these interactions may lead to improving the yield of economically important crops, as well as diminish the impact of chemical fertilizers on the environment by using nitrogen provided by BNF.

### General structure of lipopolysaccharides

**Figure 1:**
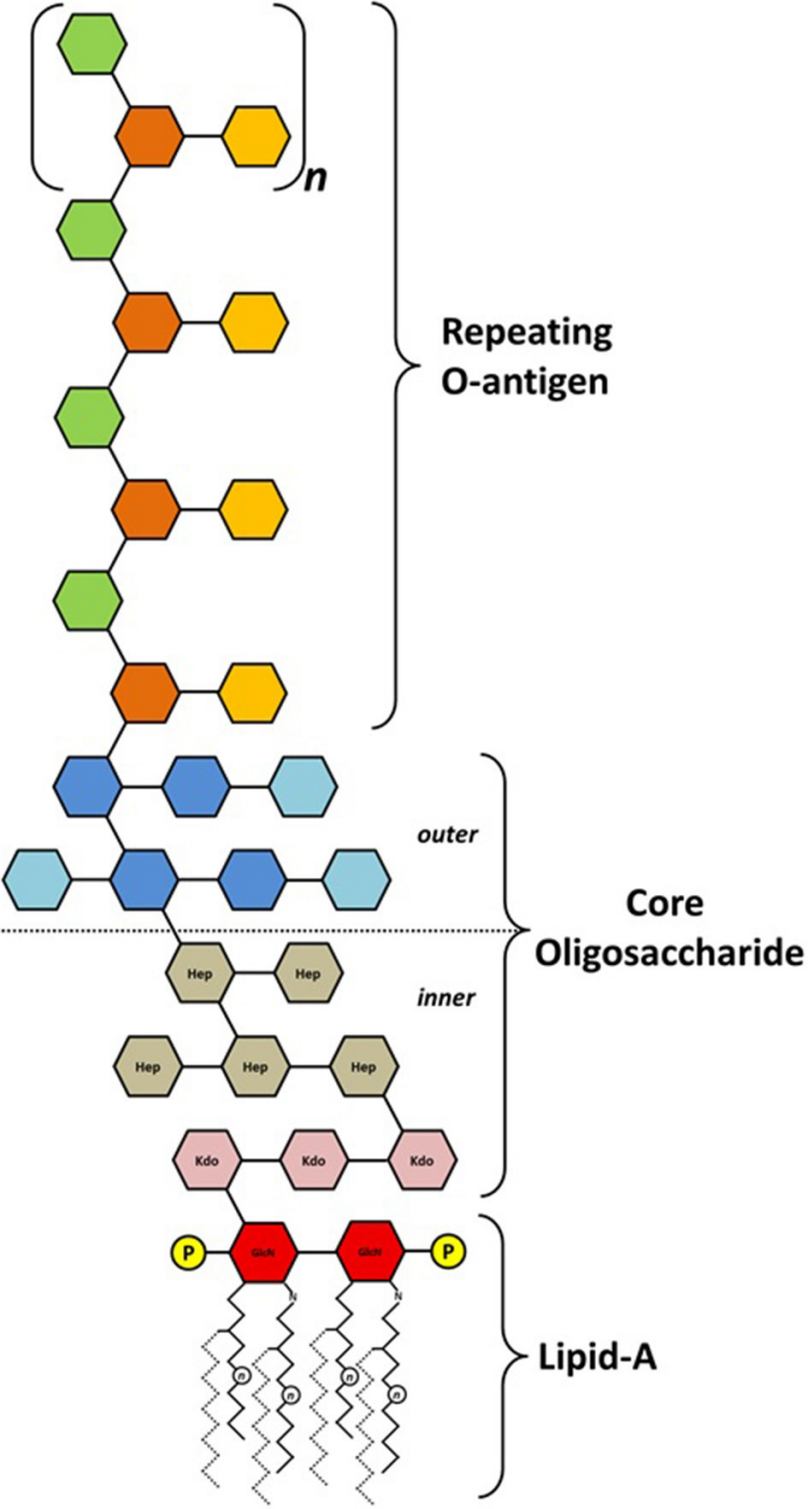
**Schematic representation of the general structure found on lipopolysaccharides**. Fatty acid chain length (*n*) and position may vary greatly among different species (secondary fatty acids shown as dotted lines). Phosphate substitutions (P) are commonly found at C1 and C4′ of both GlcN (2-amino-2-deoxy-D-glucose) units that form the lipid-A moiety. Phosphate substitutions may also be found attached to core or O-antigen units. Kdo (3-deoxy-D-manno-octulosonic acid) and Hep (D-glycero-D-mannoheptose) are most commonly found on the inner core structure, but other monosaccharides may occur.

Most Gram-negative bacteria possess LPS as the major component of the outer membrane. Typically, LPS consist of an oligo- or polysaccharide portion, respectively the core and the O-antigen moiety, anchored in the outer leaflet of the bacterium external membrane by a hydrophobic moiety named lipid-A. The latter is structurally conserved among different classes of bacteria, being formed by two units of 2-amino-2-deoxy-D-glucose (GlcN) linked by a β-(1→6) glycosidic bond and phosphorylated at positions 1 and 4′ (Zähringer et al., 1999; Trent, 2004; Wang and Quinn, 2010). Long-chain acyl groups are found either esterifying free hydroxyl groups or N-linked as amide-type substitutions on C2 of both GlcN units (Trent et al., 2006; Raetz et al., 2007). The oligosaccharide core is usually bound to the lipid-A by a Kdo unit (3-deoxy-D-manno-octulosonic acid) linked at C6 of one of the GlcN units (Raetz and Whitfield, 2002). The core varies in monosaccharide composition, but the presence of Kdo (or its derivative, Ko—D-glycero-D-talo-octulosonic acid) is almost mandatory. Some species contain D-glycero-D-mannoheptose (D,D-Hep) alone, or in combination with the most commonly found L,D-Hep, while others may have a diversity of hexopyranoses and aminosugars (Zähringer et al., 1999; Holst, 2011). Within a genus or family the structure of the inner core tends to be conserved, and the fact that distantly related bacteria share structural features is a reflection of the importance of the core in outer membrane integrity (Raetz and Whitfield, 2002). The outermost part of the LPS, the polysaccharide chain or O-antigen, lies in the interface between the bacterium and its surrounding environment, and is where the most structural heterogeneity is found. The enormous structural diversity of O-antigens lies on monosaccharide composition, glycosidic linkage position, size of repeating unit, and chain length, as well as on non-carbohydrate substitutions that may occur (Lerouge and Vanderleyden, 2001; Raetz and Whitfield, 2002). O-antigen modifications seem to play and important role at several stages of the infection process during plant-microbe interactions, including adherence, bypassing or overcoming host defenses, and establishing and maintaining intercellular communication (Knirel, 2011). Figure 1 shows a schematic model of the general structure found for LPS in Gram-negative bacteria. The complexity of LPS reflects the difficulties encountered to determine their fine structures. In many cases, only the structure of the predominant polysaccharide backbone is known. LPS extraction from bacterial cultures may also be affected by culture age and growth condition. In the case of plant-associated bacteria, culture conditions may be inadequate in order to observe the LPS present during interaction.

### Lipopolysaccharides in rhizobiaceae

**Figure 2:**
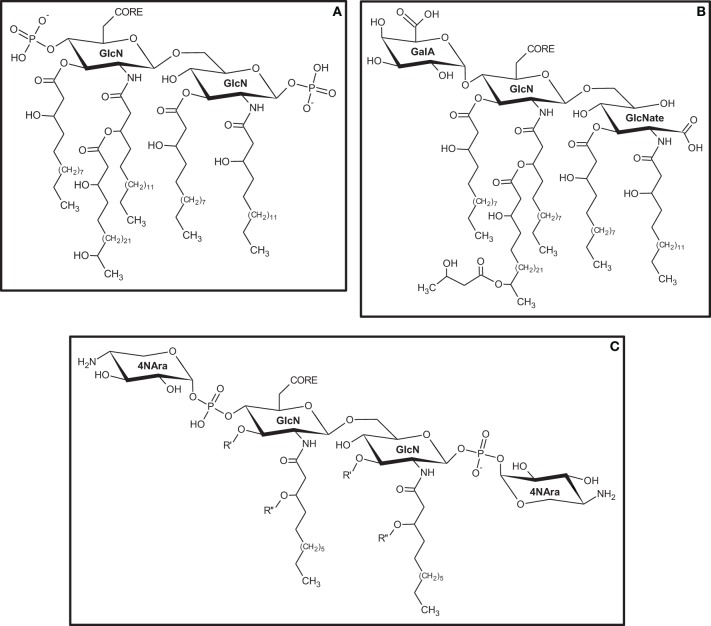
**Structural variations found on the lipid-A portion of LPS produced by diazotrophic bacteria. (A)** *Sinorhizobium meliloti*; **(B)** *Rhizobium etli* CE3; **(C)** *Herbaspirillum seropedicae* SMR1. Primary (R′) and secondary (R″) ester-linked fatty acids were not determined for *H. seropedicae* SMR1. GlcN, 2-amino-2-deoxy-D-glucose; GalA, Galacturonic Acid; GlcNate, N-acetyl-Gluconate; 4NAra, 4-deoxy-4-amino-Arabinose.

Among all diazotrophic bacteria, those belonging to the family Rhizobiaceae have certainly the greater number of species studied in regards to their LPS. Extensive work has been done on structural characterization, biosynthesis and involvement of LPS during Rhizobia-legume interaction (Carlson et al., 1999, 2010; Price, 1999; Noel and Duelli, 2000; Fraysse et al., 2003; Kesawat et al., 2009). Lipid and monosaccharide composition in LPS found for Rhizobiaceae vary considerably, but the basic architecture for this molecule is conserved (Kannenberg et al., 1998). The LPS produced by *Rhizobium etli*, strain CE3, and *R. leguminosarum* have the same basic lipid-A backbone. Instead of the typical GlcN disaccharide, both structures are formed by a trisaccharide containing GlcN, GalA, and GlcNate (gluconate) (1:1:1) (Carlson et al., 1999) (Figure 2A). In this case, the phosphate in position 4′ is replaced by a galacturonic acid unit, and both GlcN and GlcNate are N-acylated at C2 and O-acylated at C3 by β-hydroxy-fatty acids of different chain length (Bhat et al., 1994). Most lipid-A structures found in Rhizobiaceae, including *R. etli*, have very-long-chain fatty acids such as 27-hydroxyoctacosanoic acid (27-OH-C_28:0_) (Hollingsworth and Carlson, 1989; Kannenberg et al., 1998) (Figure 2B). The inner core of *R. etli* CE3 is formed by a complex highly-branched octasaccharide containing Kdo, Gal, GalA, and Man, while the outer core that binds the O-antigen has Fuc, Man, and QuiNAc (N-acetyl-quinovosamine) (Forsberg and Carlson, 1998). Despite the structural variations found in the O-antigen, the presence of deoxy-hexoses, methylated hexoses, 6-deoxy-amino-sugars, and N-methyl-6-deoxysugars is common together with the presence of acetyl substituents in the structure (Schnaitman and Klena, 1993). The O-antigen of the LPS described for *R. etli* CE3 has a trisaccharide repeating unit on its terminal portion formed by GlcA*p*, Fuc*p*, and 3Me-6dTal*p* (3-methyl-6-deoxy-talose). A cap unit of 2,3,4-tri-O-metyl-fucose is also found as non-reducing terminal (Bhat and Carlson, 1992; Forsberg and Carlson, 1998; Forsberg et al., 2000).

The nodulation process during symbiosis of rhizobia with legumes seems to be affected by the presence of truncated LPS or by the complete lack of these molecules (Carlson et al., 1995). Genes related to LPS expression and biosynthesis are modulated during symbiosis, and LPS structures are modified during the transition of free-living cells to nodule bacteroids (Broughton et al., 2006). These changes may be induced by plant extracts, and especially by flavonoids (Duelli and Noel, 1997). Mutants of rhizobia deficient in LPS biosynthesis remain on the infection thread during the early stages of nodulogenesis and are unable to complete cellular differentiation into mature nitrogen-fixing bacteroids (Noel et al., 1986; Campbell et al., 2002; Broughton et al., 2006). Mutants of *R. etli* that produce truncated LPS structures have promoted the growth of deformed nodules without the ability to fix nitrogen (Noel and Duelli, 2000). It has been proposed that LPS in rhizobia are not involved in the early stages of symbiosis (attachment, root hair curling and infection thread development), but have a central role in maintaining viable differentiated cells once de nodules are formed (Kannenberg et al., 1998; Noel et al., 2000). Furthermore, bacteroids of *Rhizobium leguminosarum* found inside the nodules of some legumes show drastic alterations in their LPS structures in comparison to the structures found for the non-differentiated cells (Goosen-Deroo et al., 1991; Kannenberg and Brewin, 1994). Bacteroids of *R. etli* and *Sinorhizobium meliloti* found in nodules of their respective plant hosts have structural differences in the O-antigen of their LPS structures similar to those found when these bacteria are cultivated in low levels of oxygen and low pH, indicating that changes in LPS structure may be due to physiological conditions to which they are exposed (Tao et al., 1992; Kannenberg et al., 1998; Reuhs et al., 1999). These data indicate that the degree of structural alterations on rhizobial LPS influence the chances of bacteroid survival and guarantees the development of an adequate nitrogen-fixing nodule on the plant host (Carlson et al., 1995).

### Lipopolysaccharides in associative and endophytic diazotrophs

Other than nodulating rhizobia, diazotrophic bacteria are also found associated with roots and rizosphere, and even inside plant tissues. This now well-known class of nitrogen-fixing bacteria, capable of establishing endophytic associations with economically important cereals and forage grasses, such as wheat, rice, sugar-cane, and maize, has been investigated in recent years with regards to their LPS structures and function during the infection process. To what concerns the structure of LPS during plant-bacterium interactions, some reports have shown that different portions of these molecules may be involved in different stages of the infection process. In *Pseudomonas syringae*, the loss or alteration of the O-antigen structure is related to an impaired virulence (Smith et al., 1994). Some works have reported the role of LPS in the adhesion process of *Agrobacterium tumefaciens* to their host cells (Pueppke, 1984; Matthysse, 1986). Mutants of this bacterium that produce LPS with an altered core structure but that maintain a non-defective o-antigen are still able to attach normally to carrot root cells (Metts et al., 1991), showing that the total structure of the LPS is not necessary to the process. The LPS produced by several strains of *Herbaspirillum* was analyzed by Serrato and coworkers (Serrato et al., 2010) showing that the LPS produced by *H. seropedicae* SmR1 was different in monosaccharide and fatty acid composition when compared to other strains. Later, the structure of the lipid-A portion of the LPS isolated from strain SmR1 was determined as having a typical β-(1→6)-linked GlcN disaccharide backbone, both units phosphorylated and decorated with units of 4-deoxy-4-amino-arabinose (4NAra) (Serrato et al., 2012) (Figure 2C). Alterations in the structure of the LPS where observed when genes related to the biosynthesis of rhamnose where knocked out in *H. seropedicae*. The LPS of both *rfb*B^−^ and *rfb*C^−^ mutants lack the entire o-antigen portion and both 4NAra units in the lipid-A. The main effect observed for both mutants is the impaired ability to colonize internal tissues of maize root (Balsanelli et al., 2010). More recently, it has been proposed that N-acetyl-glucosamine (GlcNAc) units found in the o-antigen of *H. seropedicae* LPS structure are responsible for mediating the interaction with lectins found in the root cells (Balsanelli et al., 2013).

A number of other associative nitrogen-fixing bacteria have been studied with regards to their LPS. In *Azospirillum brasilense*, the structure of the o-antigen is linear rhamnan where every unit is found as D-Rha (Fedonenko et al., 2002). Immunochemical and structural characteristics of the LPS of *A. brasilense* are also reported (Konnova et al., 2006). Another species, *A. lipoferum*, has an o-antigen backbone of α-L-Rha with a branching β-D-Glc unit (Fedonenko et al., 2008), while strain Sp59b presents a very distinct structure formed by a backbone of α/β-D-Gal*p*, branched by a tetrasaccharide containing α-L-Rha*p* and β-D-Man*p* (3:1) (Fedonenko et al., 2005). The lipid-A portion of *A. lipoferum* was described to have two β-(1→6)-linked D-GlcN units but lacks phosphate residues. Moreover, the reducing end of the backbone is found α-linked with a D-galacturonic acid unit (Choma and Komaiecka, 2008). Recent findings on the structure of o-antigen from strain SR80 of *A. brasilense* have shown that two distinct oligosaccharide repeating units are found, a trisaccharide containing D-Rha, L-Fuc, and D-Xyl (1:1:1 molar ratio respectively), and a tetrasaccharide containing D-GalNAc, L-Fuc and D-Gal (1:1:2) (Sigida et al., 2013a). Structural variations for the LPS of other strains of *A. brasilense* include the presence of 3-O-methyl-D-rhamnose units (strain Jm6B2) (Boyko et al., 2012) and 2-O-methyl-D-rhamnose (Strain Sp7) (Sigida et al., 2013b). The importance of LPS in the *Azospirillum*-plant association has been reported (Skvortsov and Ignatov, 1998; Bashan et al., 2004), but the actual role of LPS in molecular communication is yet to be understood.

A comparative analysis performed in six different strains of *Gluconacetobacter diazotrophicus* has shown a great structural variability within this species (Fontaine et al., 1995). However, the structure described for the O-antigens of *G. diazotrophicus* is similar to that previously described for some other alpha-proteobacteria, except for the presence of 2-O-substituted ribofuranose units (Previato et al., 1997). Diazotrophs of the beta-proteobacterium class have shown very distinct structures, in many cases rare and uncommon monosaccharide units are found. The presence of 3,6-dideoxy-4-C-(4′-hydroxyethyl)-D-xyloheptose, or yersiniose (YerA), has been described in the structure of the EPS produced by *Burkholderia brasiliensis* (Mattos et al., 2005). The O-antigen of *Ralstonia picketti* has shown to have units of BacNAc (4-acetamide-2-amino-2,4,6-trideoxy-D-glucose) in its structure (Vinogradov et al., 2004).

## Conclusions

BNF performed by diazotrophic bacteria has been extensively studied over the past decades, as have the symbiotic and associative processes that allow these microorganisms to invade plant tissues and deliver ammonia together with other growth-promoting substances. Even though the role of glycans and glycoconjugates, such as LPS, have been determined for some species during the infection and colonization process with their plant hosts, there are several gaps in the process that are poorly understood and require more investigation. The recent availability of numerous nitrogen-fixing bacteria genome sequences, allied to the chemical, and structural characterization of LPS, offer the tools to determine the functional aspects that these molecules play during the plant-diazotroph molecular interaction.

### Conflict of interest statement

The author declares that the research was conducted in the absence of any commercial or financial relationships that could be construed as a potential conflict of interest.
